# Technology-Based Solutions to Improve Management of COVID-19: A Call for More Utilization in Iran

**DOI:** 10.18502/ijph.v49i8.3912

**Published:** 2020-08

**Authors:** Mehdi HASSANIAZAD, Marjan GHAZISAEEDI, Tayebeh BANIASADI

**Affiliations:** 1. Infectious and Tropical Diseases Research Center, Hormozgan Health Institute, Hormozgan University of Medical Sciences, Bandar Abbas, Iran; 2. Department of Health Information Management, School of Allied Medical Sciences, Tehran University of Medical Sciences, Tehran, Iran

## Dear Editor-in-Chief

Coronavirus Disease 2019 (COVID-19) pandemic as a new infectious disease has forced all healthcare systems to utilize technology-based services more than ever before (1). With previous successful experiences of using health-related technologies in the management of infectious respiratory epidemics such as SARS and MERS, it can be expected that most of them be useful in this new type of epidemic as well. Management of infectious disease generally contains prevention, control, diagnosis, isolation, treatment, and follow-up care phases, which eHealth in each of these phases can potentially be effective.

In the phases of control, prevention, and screening, supportive technologies such as virtual electronic educations, web sites, and mobile apps (2) help to the promotion of knowledge and improvement of self-care skills in people. Tele-visits and chatbots as virtual agents (3) are other services maybe support to early detect people with disease symptoms for faster act then prevent unintended outcomes. In this time, COVID-19 tracking systems based on GIS technologies and notification service can support the process of surveillance (4). Robots are another emerging technology that their potential roles during the COVID-19 outbreak are becoming increasingly clear. The robots have the capabilities for disinfection and handling of contaminated waste, medications and food delivery, and measurement of vital signs (5).

Early diagnosis of suspected or symptomatic COVID-19 patients for rapid treatment and timely isolation is important. Decision support systems based on artificial intelligence through useful algorithms can facilitate the faster diagnosis of COVID-19 cases as well as planning for proper treatment of the affected individuals. The researchers with the machine learning approach were able to develop a deep learning model from chest CT images in order to accurately distinguish COVID-19 from other types of pneumonia (6).

The remote surveillance of home-isolated patients for supporting care and tracking adherence to related protocols through mobile apps can be an effective solution.

In the care phase, continuous monitoring of inpatients can be done by emerging technologies like monitoring devices and wearable smart sensors (7) since they enable timely diagnosis of variation of the clinical condition and critical symptoms. It leads to minimizing face-to-face contact with patients then reducing the healthcare provider’s exposure.

Moreover, combinations of electronic health records (EHR), computerized provider order entry (CPOE) and clinical decision support system (CDSS) could support the clinical needs of a health system in managing the COVID-19 pandemic. The operational dashboard with real-time data on the number of patients tested, test results, intensive care unit and ventilator use and availability, and volume of ambulatory visits are effective in COVID-19 outbreak management (8).

The use of telemedicine services such as remote consultation (9) is helpful, especially when physicians do not have a physical presence or specialists are insufficient in number. Furthermore, teleradiology helps to speed up diagnostic and therapeutic processes (10).

Creating a platform for electronic follow-up of discharged COVID-19 patients (1) by any digital technology solution (e.g. mobile apps) for tracking recurrence of symptoms and the possible risk factors of subsequent readmission is necessary. The automatic interactive voice response systems or chatbots can cover the lack of human resources in the follow-up and recovery setting.

Generally, integrated informatics technologies despite some potential challenges and limitations, facilitate processes related to patient care management that lead to the optimization of workflow and services delivery. In conclusion, planning for investing in health informatics technologies can bring great benefits to the era of COVID-19 and similar disease pandemics in the future. Thus we call for further applied research and investments in Iran.
